# A novel NSAID derivative, phospho-ibuprofen, prevents AOM-induced colon cancer in rats

**DOI:** 10.3892/ijo.2012.1756

**Published:** 2012-12-28

**Authors:** NENGTAI OUYANG, PING JI, JENNIE L. WILLIAMS

**Affiliations:** Division of Cancer Prevention, Department of Medicine, Stony Brook University, Stony Brook, NY 11794-8175, USA

**Keywords:** non-steroid anti-inflammatory drug, colon cancer, NF-κB, azoxymethane, COX-2, β-catenin

## Abstract

The cancer chemopreventive properties and gastrointestinal toxicity of ibuprofen are well documented. Modification of existing NSAIDs has improved on the chemopreventive efficacy of this agent and reduced its toxicity. In this study, ibuprofen and a modified derivative (phospho-modified ibuprofen or p-ibuprofen) were used in a chemically induced model of colon cancer. Fisher 344 rats were injected with azoxymethane then treated with either ibuprofen (500 ppm) or p-ibuprofen (900 ppm) for 20 weeks to observe aberrant crypt foci (ACF) or 40 weeks to evaluate tumor incidence and multiplicity. β-catenin and p65 were measured in colonic tissues by immunofluorescence staining. Equal molar doses of ibuprofen (75 and 670 mg/kg) and p-ibuprofen (135 and 1,215 mg/kg) were administered to rats for 7 days to assess acute toxicity. The *in vitro* effect of p-ibuprofen on COX-2 and PGE_2_ synthesis, β-catenin expression and NF-κB activity were examined in RAW 264.7 macrophage and HCT 116 colon cancer cells. At week 20, p-ibuprofen and ibuprofen significantly reduced the multiplicity of ACF compared with control (p<0.05); 31.2 and 37.9%, respectively. At week 40, p-ibuprofen and ibuprofen reduced the multiplicity of colon tumors compared with control (p<0.01) by 47.2 and 56.6%, respectively. Equal molar concentrations of ibuprofen (670 mg/kg) and p-ibuprofen (1,215 mg/kg) resulted in stomach ulceration in 85.7% (6 out of 7) and 14.3% (1 out of 7) of rats, respectively, with p<0.01. Immunofluoresence staining and western blot analysis demonstrated that both ibuprofen and p-ibuprofen suppressed β-catenin nuclear translocation in colon cancer cells. In addition, p-ibuprofen but not ibuprofen inhibited NF-κB activation in colon cancer cells. Collectively, these results suggest that p-ibuprofen is a potential effective novel drug for long-term use in colon cancer prevention.

## Introduction

Numerous animal studies and clinical trials in cancer have shown that ibuprofen reduces the incidence of and mortality from cancer (1–3). However, adverse effects, such as increased gastrointestinal (GI) ulceration, limit its potential for long-term use. To reduce this side effect, different modifications of ibuprofen have been sythesized and evaluated. These modifications include guiacol ester (4), alkyl- or thio-ester (5), diethylcarbonate (6), 2-formylphenyl ester (7), N-hydroxymethyl-succinimide (8), β-D-glucopyranoside (9), polymerized-2-hydroxyethylmethacrylate (10), PEG1000-linked chondroitin (11), α-methyl, ethyl and propyl glucopyranosides (12), cysteamide (13), L-cysteine ethyl ester (14) and NO-donating moieties (15–17). Although most of these modifications did result in a reduction of GI side effects, only a decreased or similar effect of anti-inflammation or anticancer activity was observed when compared with the parent compound ibuprofen. Recently we developed a phospho-butanol-modified ibuprofen (designated p-ibuprofen, hereinafter), which showed promising increased anticancer activity *in vitro* and in xenograft tumor models (18–20), elevated anti-inflammation in an arthritis rat model (21), and reduced GI toxic side effects. Xie *et al*(20) found that p-ibuprofen is minimally metabolized by cultured cells, but extensively metabolized by mouse liver microsomes, undergoing regioselective oxidation to produce 1-OH-p-ibuprofen and carboxyl-p-ibuprofen, which can be hydrolyzed to the parent metabolites, 1-OH-ibuprofen and carboxyl-ibuprofen, respectively. These results indicate that the anticancer effect of p-ibuprofen may be different between *in vitro* and *in vivo* situations. Therefore, additional *in vivo* studies are necessary to evaluate the chemopreventive effect of p-ibuprofen before it can be considered for human clinical trials.

Both COX-2 dependent and independent pathways may be involved in the mechanism by which NSAIDs prevent cancer. It remains to be fully elucidated whether p-ibuprofen suppresses cancer growth by the COX-2 pathway or COX-2 independent pathways. Previous studies (19–21) have suggested that oxidative stress mediated apoptosis, reduced inflammatory cytokines, and inhibition of NF-κB activation are involved in the mechanism of its action. Wong *et al*(22) reported that both p-ibuprofen and ibuprofen can be hydrolyzed by carboxylesterases in the liver, and that the integrity of the drug is critical for anticancer activity. p-Ibuprofen’s reduced GI toxicity may be a result of the modification of the phospho-group (with the COOH-group), which is known to account for the GI toxicity of the conventional NSAIDs (23).

In this study, we tested the chemopreventive effect of p-ibuprofen in a long-term use scenario using a chemical-induced colon cancer model in rats. The acute and chronic toxicities of p-ibuprofen were evaluated in rats. In additon, the effect of p-ibuprofen on COX-2-dependent and -independent pathways, including β-catening and NF-κB pathways, were studied *in vitro*.

## Materials and methods

### Drugs

**Figure 1.**
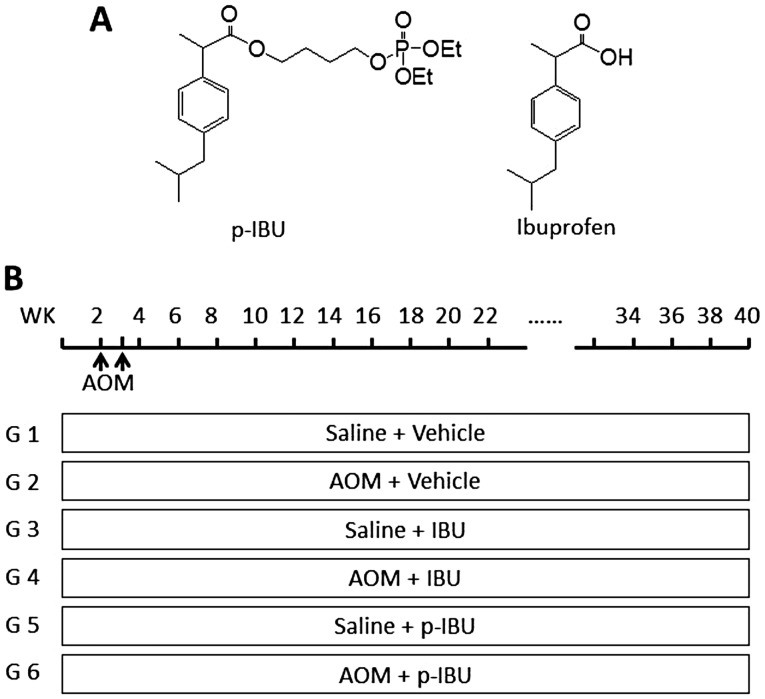
Chemical structure of p-IBU and design of *in vivo* experiment. (A) The chemical structures of p-IBU and parent molecule IBU are presented in the top panel. (B) As described in Materials and methods, animals were placed on a control diet or study diet (food supplemented with of IBU or equal molar concentration of p-IBU) for 10 days prior to subcutaneous injection with 15 mg/kg AOM, once a week for two weeks (bottom panel).

Ibuprofen was purchased from Sigma (St. Louis, MO). Phospho-butanol-ibuprofen was purchased from Chem-Master International Inc. (East Setauket, NY). The chemical structure is shown in Fig. 1A. The purity of synthetized drug p-ibuprofen is >99%.

### Animal model and treatments

Fisher 344 male rats (135) (Harlan Sprague Dawley, Indianapolis, IN), 3–4 weeks old with an initial average body weight of 90 g, were acclimated for 1 week, divided into 6 groups: groups 1, 3 and 5 were given saline as control, 15 rats per group; and groups 2, 4 and 6 were given carcinogen, 30 rats per group. Animals received either saline or carcinogen (azoxymethane, AOM, at 15 mg/kg) by subcutaneous injection, once a week for two weeks. Drug administration was also initiated in rats via diet 10 days before subcutaneous injections: vehicle for groups 1 and 2; ibuprofen, 500 ppm, for groups 3 and 4; and phosphoibuprofen (p-ibuprofen, the same hereinafter), 900 ppm (equal molar dose with ibuprofen), for groups 5 and 6. All drugs were administered up until the end of the experiment as shown in Fig. 1B. Animals were housed and maintained according to the approved standards of Stony Brook University Institutional Animal Care and Use Committee. Animals were housed two per plastic cage with sawdust bedding and were kept under standard laboratory conditions (room temperature, 22±2°C; relative humidity, 50±5%; light/dark cycle 12/12 h). All animals had access to food and tap water *ad libitum*. Rats were observed daily and weighed once a week. Half of the rats from each group were euthanized at week 20 and analyzed for aberrant crypt foci (ACF). Blood was collected for pharmacokinetic (PK) analysis. The remaining rats were euthanized at week 40 and colons were dissected and analyzed for aberrant crypt foci (ACF) and tumors. Heart, lung, liver, stomach and kidney were collected from animals in the control groups and fixed in buffered-formalin for histological analysis of potential toxic effects.

### ACF analysis

**Figure 2.**
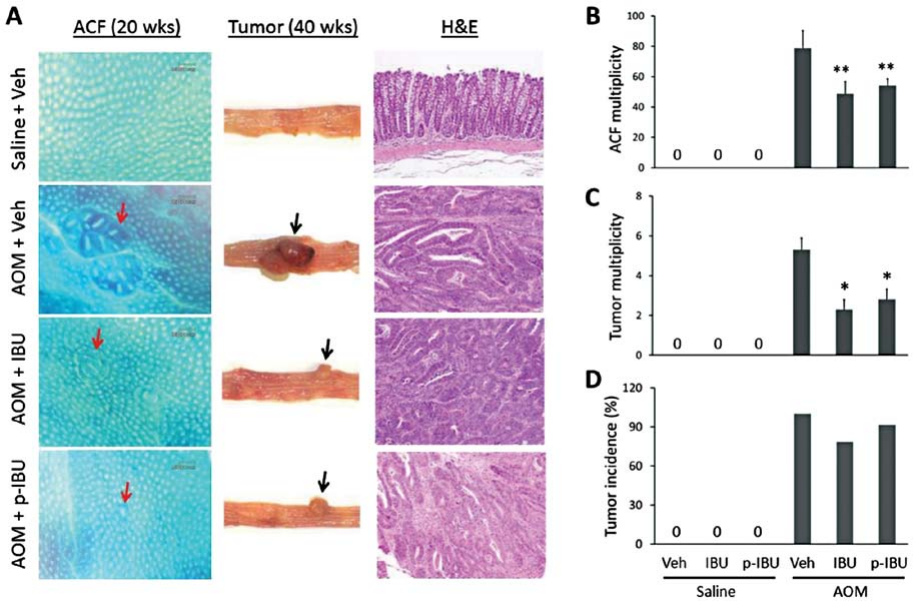
p-IBU inhibits AOM-induced colonic ACF multiplicity and tumor multiplicity in rats. (A) The number of ACFs or tumors per animal was counted in various treatment groups as indicated above. The results show that the number of ACFs and tumors in the colon of AOM treated rats was greatly reduced following treatment with IBU and p-IBU (^**^p<0.05 or ^*^p<0:01, Student’s t-test). Dissected colon in descending order: saline + control diet showing no tumors; AOM + control diet showing the presence of tumors (arrows); AOM + diet supplemented with IBU showing reduced number of tumors and; AOM + diet supplemented with p-IBU showing reduced number of tumors. (B) ACF multiplicity; (C) tumor multiplicity; and (D) tumor incidence.

For animals sacrificed at week 20, ACF were counted in colon tissues as previously described (24). Briefly, the colons were removed, rinsed with ice-cold phosphate-buffered saline (PBS), placed on filter paper, opened longitudinally, and fixed in 10% buffered formalin for 24 h. Then colon tissues were stained with 0.2% methylene blue for 3 to 5 min. The number of ACF per colon was determined by microscopic examination. ACF were distinguished from surrounding normal crypts by increased size, thickened epithelial cell lining, and enlarged cryptal area relative to surrounding normal crypts as shown in Fig. 2A.

### Tumor analysis

At week 40, the remaining animals were sacrificed by CO_2_ asphyxiation. Colons were removed, opened longitudinally, and rinsed with PBS. Tumors were counted and both long-diameter by short-diameter were measured to calculate tumor size. After measurements were recorded, one half of each colon sample was frozen in liquid nitrogen and stored at −80°C for further analyses. The remaining half of each colon was fixed in buffered formalin for histopathology processing. Briefly, colon tissue with tumors were equally cut into 10 pieces, and embedded in paraffin blocks. Sections (4 *μ*m) were stained with hematoxylin and eosin to determine histopathology by pathologist, or stained by immunohistochemistry and immunofluorescence for mechanism study.

### Toxicity

Thirty-five Fisher 344 rats (Harlan Sprague Dawley), male, 8–9 weeks old, were divided into 5 groups (7 rats per group) and treated with the following: group 1, vehicle; group 2, ibuprofen 75 mg/kg of body weight; group 3, ibuprofen 670 mg/kg; group 4, p-ibuprofen 135 mg/kg; and group 5, p-ibuprofen 1,215 mg/kg. The drugs were administered by gavage, once a day for 7 days. Animals were housed under standard conditions and euthanized by CO_2_ asphyxiation 1 h after final drug administration. Blood was collected for PK studies. Stomach, small intestine and colon were checked for ulcers under magnification lens then fixed with 10% buffered formalin for histology. Heart, liver, lung and kidney were collected for toxicity analyses.

### Pharmacokinetics and HPLC analysis

The blood samples collected from animals sacrificed at week 20 and from the toxicity study with low dose treatment were used for the PK study. Briefly, ibuprofen and its metabolites were extracted by adding a 2-fold volume of acetonitrile. After centrifugation for 10 min at 5,000 × g, the supernatants were subjected to HPLC analysis. The HPLC system consisted of a Waters Alliance 2695 Separations Module equipped with a Waters 2998 photodiode array detector (220 nm) (Waters, Milford, MA) and a Thermo BDS Hypersil C18 column (150×4.6 mm, particle size 3 *μ*m) (Thermo Fisher Scientific, Waltham, MA). The mobile phase followed a gradient between buffer A [formic acid, acetonitrile, H_2_O (95:4.9: 0.1 v/v/v)] and buffer B (acetonitrile).

### Prostaglandin E2 (PGE_2_) and COX-2 in vitro

To evaluate the inhibitory effect of ibuprofen or p-ibuprofen on COX-2, RAW 264.7 macrophages were pre-treated with ibuprofen or p-ibuprofen, 130 *μ*M, for 12 h, then incubated with LPS, 100 ng/ml, overnight. The cells were collected for COX-2 measurement and analyzed by western blotting. Levels of PGE_2_ in cell culture media were determined using a commercially available immunoassay kit according to the manufacturer’s instructions. Briefly, 1.5×10^6^ Raw 264.7 macrophages were pre-incubated with ibuprofen or p-ibuprofen, 130 *μ*M, for 12 h, followed by LPS (100 ng/ml) overnight. The cultured media were collected to measure PGE_2_ level using an ELISA kit (Cayman Chemical, Ann Arbor, MI).

### Immunofluorescent double staining

Paraffin-embedded sections were deparaffinized, rehydrated and microwave heated for 15 min in 0.01 mol/l citrate buffer (pH 6.0) for antigen retrieval. Tissue sections were then incubated with 5% donkey serum for 30 min, then treated with primary antibodies, rabbit anti-phospho-p65, ser276 (Cell Signaling, Danvers, MA) and mouse anti-β-catenin (Millipore, Temecula, CA) or control IgG, at 4°C overnight. After washing with PBS 3×5 min, the secondary donkey anti-mouse IgG and donkey anti-rabbit IgG conjugated with fluorescents were added and incubated at room temperature for 1 h. Slides were washed thrice with PBS, mounted with media and observed under a fluorescence microscope.

### Cell culture

RAW 264.7 macrophage (mouse leukemic moncyte) and HCT 116 colon cancer cell lines were purchased from American Type Culture Collection (Manassas, VA) and cultured in DMEM or RPMI-1640 medium, respectively.

### Western blot analysis

Whole cell extracts were obtained by lysing cells in RIPA buffer [50 mM Tris-HCl (pH 7.4),150 mM NaCl, 1 mM Na_2_EDTA, 1 mM phenylmethylsulfonyl fluoride (PMSF), 1% NP-40, 0,25% sodium deoxycholate and Protease Inhibitor Cocktail 2 (Sigma-Aldrich)]. Cytoplasmic and nuclear extracts were prepared following a standard protocol (25); trypsinized cells were suspended in lysis buffer to which NP-40 was added at a subsequent step (the supernatant fraction represented the cytoplasmic extract); nuclei were washed and centrifuged, followed by resuspension in extraction buffer and pelleting. Protein extracts were analyzed by a well-established standard western blot procedure (26). Rabbit anti-COX-2 antibody was purchased from Cayman Chemical. Other primary antibodies are indicated in the immunofluorescence staining.

### Statistical analysis

Data are expressed as mean ± SEM and analyzed with ANOVA. P≤0.05 was considered statistically significant.

## Results

### p-Ibuprofen prevents AOM-induced colon cancer

AOM-induced ACF at week 20 and colon tumors at week 40 as shown in Fig. 2A. Histology of tumors shows well-differentiated adenocarcinoma. As shown in Table I and Fig. 2B, compared to vehicle treatment, both p-ibuprofen or ibuprofen significantly reduced the multiplicity of ACF by 31.2% (54.2±4.4 vs. 78.8±11.6, p<0.05), or 37.9% (48.9±7.9 vs. 78.8±11.6, p<0.05), respectively. However, no difference was observed between groups treated with p-ibuprofen or ibuprofen (p>0.05). Similarly, as shown in Table I and Fig. 2C, at week 40 treatment with either p-ibuprofen or ibuprofen reduced the multiplicity of colon tumors by 47.2% (2.8±0.52 vs. 5.3±0.59, p<0.01), or 56.6% (2.3±0.49 vs. 5.3±0.59, p<0.01), respectively. Again, a significant difference (p>0.05) was not observed between groups treated with p-ibuprofen and ibuprofen (Table I and Fig. 2D). Furthermore, pathological changes were not detected in heart, lung, liver, stomach and kidney tissues. These results suggest that phospho-modification did not significantly enhance the inhibitory effect of ibuprofen in AOM-induced colon cancer in rats.

### Phospho-modification of ibuprofen reduces GI toxicity

**Figure 3.**
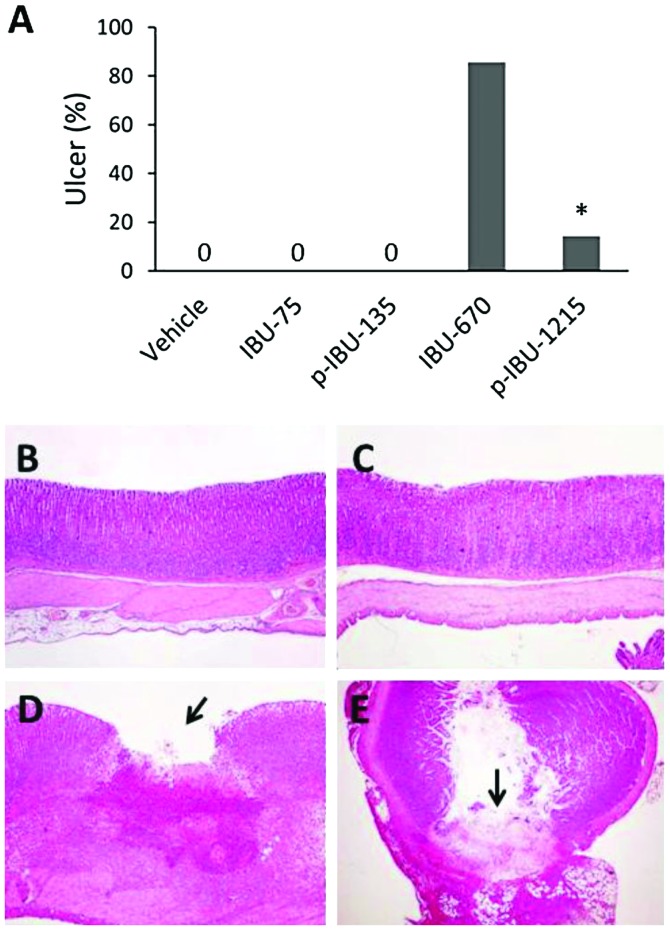
p-IBU reduces toxic GI side effect in rats. Small intestines were dissected and examined for ulceration. (A) A high dose of IBU (670 mg/kg) administered by gavage for 7 days led to stomach ulceration in 85.7% of rats; whereas, an equal molar dose of p-IBU (1,215 mg/kg) caused stomach ulceration in only 14.3% of rats (^*^p<0.01). (B) Immunohistochemical staining representative of intestines without ulceration from vehicle-treated rats; (C) representative intestines without ulceration from rats treated with low doses of IBU or p-IBU; (D) representative intestines with typical ulcerations observed with high doses of IBU or p-IBU and; (E) representative intestines with inflammation and perforation observed with high doses of IBU.

As shown in Fig. 3A, a high dose of ibuprofen (670 mg/kg) administered to rats by gavage for 7 days led to stomach ulcerations in 85.7% (6 out of 7) of rats; whereas, an equal molar dose of p-ibuprofen (1,215 mg/kg) caused stomach ulceration in only 14.3% (1 out of 7) of rats. The modification of ibuprofen with the phospho-moiety remarkably reduced its GI side-effect (p<0.01). All animals with stomach ulcers also exhibited ulcerations in the small intestine. Histologically, the typical ulcer appeared with the hiatus of mucosa and ulcerative tissue in the bottom of the ulcer (Fig. 3D). Some of the ulcers exhibited inflammation and perforation (breaking through the wall) in both stomach and intestine (Fig. 3B–E). A low dose of both p-ibuprofen and ibuprofen did not lead to ulceration in either stomach or intestine. There was no obvious pathological change in heart, liver, lung and kidney. These results demonstrate that, whereas the phospho-modification of ibuprofen did not enhance ibuprofen’s inhibitory effect on AOM-induced colon cancer in rats, p-ibuprofen significantly reduced the GI toxicity of ibuprofen.

### Pharmacokinetics

**Figure 4.**
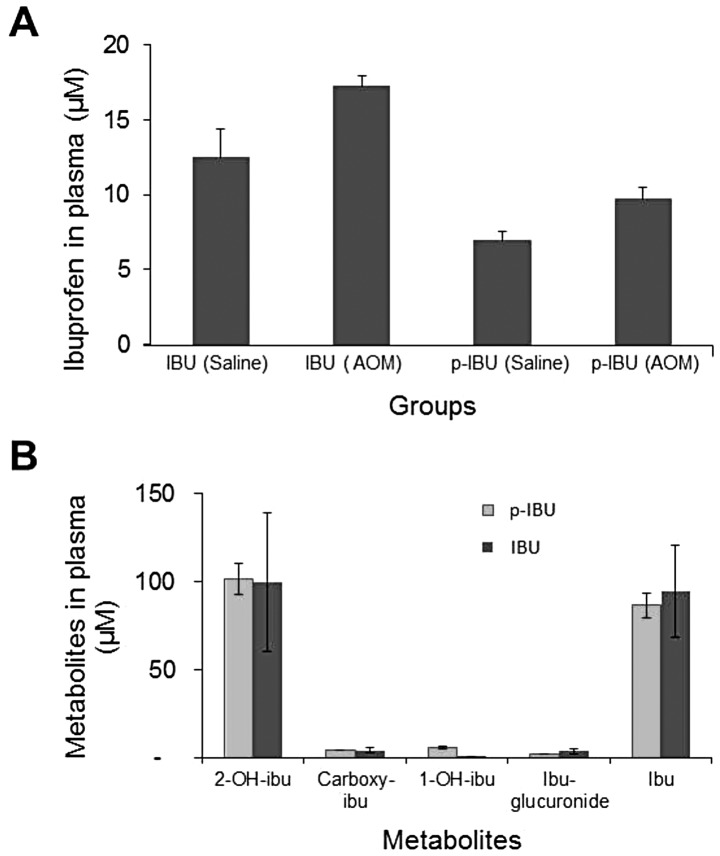
Pharmacokinetics of p-IBU. (A) Detection of IBU in the plasma of rats treated with IBU or p-IBU in the diet for 20 weeks. (B) Plasma level of the metabolites of IBU (2-OH-ibuprofen, carboxy-ibuprofen, 1-OH-ibuprofen, and ibuprofen-glucuronide). These results are comparable to levels seen in the plasma of rats on the same diet for 40 weeks or gavaged with low or high doses of the drug for 7 days.

As shown in Fig. 4A, with continuous administration of ibuprofen or p-ibuprofen in the diet for 20 weeks, ibuprofen can be detected in the plasma of both treatment groups. The plasmic level of ibuprofen in animals fed a diet with p-ibuprofen is lower than that of animals fed a diet of ibuprofen. Both diet groups (p-ibuprofen and ibuprofen) exhibited the tendency of a higher plasmic ibuprofen level in AOM-treated animals as compared to saline control animals. However, no statistical difference was noted. These trends were comparable in the case where rats were on the same diet for 40 weeks. Intact p-ibuprofen was not detectable in the plasma of animals fed a diet consisting of or gavaged with p-ibuprofen. As seen in Fig. 4B, the metabolite of ibuprofen, 2-OH-ibuprofen, was detectable at a similar level as intact ibuprofen in both groups fed a diet of p-ibuprofen (102 *μ*M) or ibuprofen (99 *μ*M). Other metabolites were detected at very low levels, including carboxy-ibuprofen (3 and 3 *μ*M), 1-OH-ibuprofen (4 *μ*M, undetectable) and ibuprofen-glucuronide (1 and 1 *μ*M); 20 weeks and 40 weeks, respectively.

### p-Ibuprofen inhibits COX-2 and PGE_2_ in macrophages

**Figure 5.**
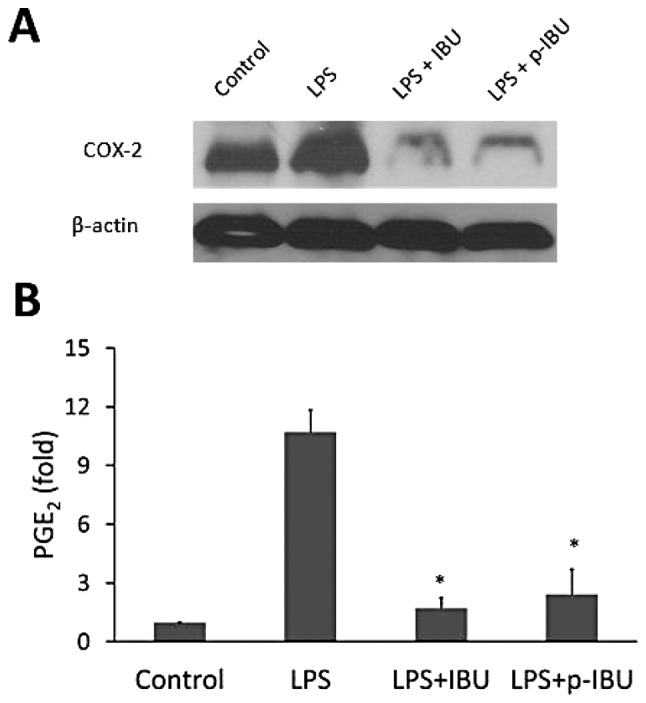
p-IBU inhibits COX-2 and PGE_2_ in macrophages. RAW 264.7 macrophages were pre-treated with IBU or p-IBU, 130 *μ*M for 12 h, then incubated overnight with LPS (100 ng/ml). (A) Western blot analysis demonstrates that induction of COX-2 levels by LPS was completely blocked by both IBU and p-IBU. (B) Evaluation by ELISA demonstrated that both IBU and p-IBU significantly suppressed PGE_2_ production (p<0.01) compared to LPS-treated control.

We evaluated the classic pathway of NSAIDs-COX-2 expression in macrophages. Our results showed that LPS highly induced COX-2 levels and that this effect was completely blocked by both ibuprofen and p-ibuprofen (Fig. 5A). These results suggest that phospho-modification did not alter the property of ibuprofen in inhibiting COX-2 expression.

As seen in Fig. 5B, both ibuprofen and p-ibuprofen significantly suppressed PGE_2_ production by 84.1% (1.7±0.5 vs. 10.7±1.1, p<0.01) and 77.4% (2.4±1.3 vs. 10.7±1.1, p<0.01) as compared to LPS-treated control, respectively. However, no significant difference between ibuprofen and p-ibuprofen (p>0.05) was observed. This result is consistent with the observed inhibition of COX-2, suggesting that phospho-modification does not decrease the ability of ibuprofen to suppress PGE_2_ synthesis.

### p-Ibuprofen suppresses β-catenin nuclear translocation and NF-κB activation

**Figure 6.**
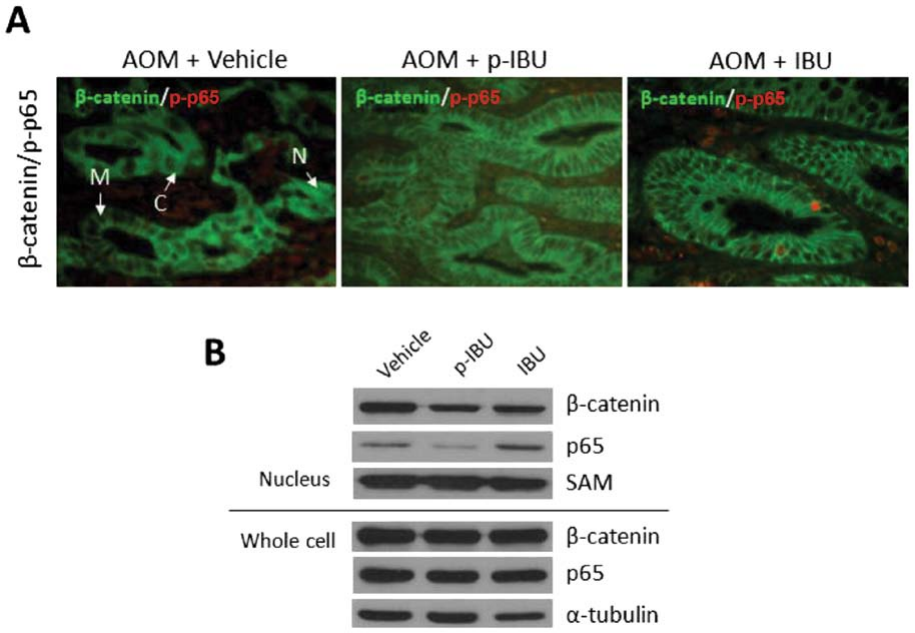
p-IBU inhibits β-catenin nuclear translocation and NF-κB activation in cancer cells. (A) Paraffin-embedded sections from rat AOM *in vivo* study (40 weeks) were prepared for immunohistochemical analysis as described in Materials and methods. p-IBU and IBU reduced AOM induced β-catenin cytoplasmic accumulation and nuclear translocation. p-IBU inhibited nuclear translocation of the NF-κB subunit p65. M, membrane; C, cytoplasmic; N, nuclear. (B) Western Blot analysis (*in vitro* analysis) using nuclear extracts of HCT116 cells demonstrated that both p-IBU and IBU markedly decreased β-catenin levels. p65 levels were reduced in cells treated with p-IBU.

As shown in Fig. 6A, exposure to the carcinogen AOM induced β-catenin cytoplasmic accumulation and nuclear translocation. These effects were reduced by both p-ibuprofen and ibuprofen. The presence of phosphorylated NF-κB subunit p65 indicates NF-κB activation. By immunohistochemistry analysis (Fig. 6A), p-ibuprofen and not ibuprofen inhibited phosphorylation of p65 (NF-κB activation). This *in vivo* observation was confirmed by an *in vitro* study. As seen in Fig. 6B, treatment with both p-ibuprofen and ibuprofen markedly decreased β-catenin levels in the nuclear extracts of HCT116 cells as compared to control (vehicle-treated). Interestingly, p-ibuprofen inhibited the nuclear translocation of the NF-κB subunit p65 (NF-κB activation), but this inhibitory effect was not observed for ibuprofen. This additional inhibitory effect in NF-κB activation may explain why the lower plasmic level of ibuprofen after treatment with p-ibuprofen exhibited a similar tumor inhibitory effect with ibuprofen-treated group.

## Discussion

The modification of existing NSAIDs is significant for developing novel drugs in cancer prevention. To date, there have been no reports indicating that modified ibuprofen possesses increased anticancer activity and reduced GI toxic side effects. For example, Shanbhag *et al*(27) modified ibuprofen by esterification and amidation with various groups. These modified agents exhibited less anti-inflammatory activity, but only ibuprofen-O(CH_2_)2N(CH_3_)2HCl and ibuprofen-NHCH_2_COOH showed decreased GI toxic side effects when compated to the parent molecule. However, our group developed a novel phospho-butanol-modified ibuprofen that exhibits a markedly higher anti-inflammatory efficacy *in vivo*(21) and anticancer activity *in vitro* and in xenograft models (19, 20) compared with its parent compound ibuprofen. Respectively, this study showed that phospho-modified ibuprofen significantly reduced the GI toxic side effect compared to the parent ibuprofen. In addition, this compound inhibited AOM-induced colonic ACF and tumor multiplicity in rats in an inhibitory manner similar to the parent compound ibuprofen. This study also shows that this modification significantly reduces the GI toxic side effect associated with the unmodified parent, ibuprofen. p-Ibuprofen inhibits AOM-induced colonic ACF and tumor multiplicity in rats with a potency that is comparable to ibuprofen. As the PK results show, ibuprofen was released into the blood of animals after administration with p-ibuprofen. However, this level is slightly lower when compared to direct ibuprofen administration. This may explain why p-ibuprofen is able to retain the anticancer properties of ibuprofen.

For decades, the mechanisms by which NSAIDs prevent cancer have focused on cyclooxygenase (COX) inhibition (28,29). Recently, COX-2 has been shown to be upregulated in various carcinomas and to have a central role in tumorigenesis (30,31). The classical NSAIDs are not selective and inhibit both COX-1 and COX-2, which results in the inhibition of prostaglandin and thromboxane synthesis and reduced inflammation. This mode of action also causes an adverse effect, irritation of the gastric mucosa, as prostaglandins are considered to have a protective role in the gastrointestinal tract. NSAIDs that have been engineered to selectively inhibit COX-2, such as celecoxib and rofecoxib, cause much less gastric irritation but may increase the risk of heart attack and thrombosis as a result of the increase of thromboxane unbalanced by prostacyclin. In the current study, phospho-modification greatly reduced the GI ulcerogenicity of ibuprofen even when a high dose (9 times regular dose) was used in rats. This was considered to result from both the reduced irritation of free carboxylic group and the inhibition of synthesis of gastrointestinal PGE_2_ and PGI_2_ (32–34). Consistent with these findings, our results also showed that, like the parent ibuprofen, p-ibuprofen retained the ability to inhibit COX-2 and PGE_2_ synthesis. We did not detect additional toxic effect of p-ibuprofen in heart, lung, liver or kidney of rats treated with both regular and high doses; thereby, suggesting that p-ibuprofen is a potential novel drug for long-term use in cancer prevention.

COX inhibition is not the only mechanism by which NSAIDs act. Increasing evidence has shown that COX-independent pathways are involved in the mechanism of action of NSAIDs, especially in cancer prevention via inhibition of cell cycle progression (35–38), induction of apoptosis (39,40), anti-angiogenesis (41,42), the mTOR signaling pathway (43), direct gene alteration (44), oxidative phosphorylation in mitochondria (45,46), the induction of nitro oxide radicals (47), and the NF-κB signaling pathway (26,48). Further investigation of COX-independent pathways is necessary in order to gain a complete understanding of the mechanism by which NSAIDs prevent cancer. In this study, we show that both ibuprofen and phospho-modified ibuprofen significantly inhibit β-catenin nuclear translocation both *in vivo* and *in vitro*. This finding is consistent with the results reported by Maier *et al*(49). Our preliminary results show that p-ibuprofen significantly inhibits NF-κB activation in a human colon cancer cell line. NF-κB, as a master transcriptional regulator of inflammatory response, may be involved in the mechanism of carcinogenesis (50,51). Normally NF-κB is bound to an inhibitor, I-κB, in the cytoplasm. To activate the signaling pathway, an I-κB kinase (IKK) is phosphorylated and activated. The activated IKK degrades the NF-κB inhibitor I-κB (canonical pathway) or p100 (non-canonical pathway) and releases p65/p50 or RelB/p52 dimers (activated subunit forms of NF-κB) to the nucleus. These proteins then regulate gene transcription for cell survival, proliferation, and inflammation. We will perform additional studies to explore how mechanistically p-ibuprofen activates NF-κB signaling pathway.

In summary, phospho-modification of ibuprofen remarkably reduces its GI toxic side effects while allowing it to retain the anti-inflammatory and anticancer activities of its parent compound. In addition to COX-2 mechanism, both ibuprofen and phospho-modified ibuprofen may inhibit the β-catenin signaling pathway and may suppress NF-κB activation in cancer cells. Taken together, these results solidify our hypothesis that p-ibuprofen is a potential effective novel drug for long-term use in colon cancer prevention.

## Figures and Tables

**Table I.** Inhibition of AOM-induced colonic ACF and tumor multiplicity in rats by p-IBU.

Diet	Control		IBU		p-IBU	
	Saline^a^	AOM^a^	Saline^a^	AOM^a^	Saline^a^	AOM^a^
20 Weeks						
Weight	447±10.60	423±9.45	416±6.11	406±118.91	420±6.97	400±8.14
ACFs	0	78.8±11.6	0	48.9±7.9	0	54.2±4.4
Tumors	0	3.3±0.72	0	1.3±0.31	0	1.7±0.42
40 Weeks						
Weight	467±7.97	434±11.19	431±15.43	432±8.84	455±5.49	458±12.33
ACFs	0	34±5.17	0	35±4.05	0	45±3.68
Tumors	0	5.3±0.59	0	2.3±0.49	0	2.8±0.52

a Average ± SE.

P-values (ACF/20 weeks): control to IBU, p<0.009; control to p-IBU, p<0.046; and IBU to p-IBU, p<0.216. P-values (tumor/40 weeks): control to IBU, p<0.002; control to p-IBU, p<0.030; and IBU to p-IBU, p<0.152.
